# *Streptococcus pneumoniae* associated hemolytic uremic syndrome in children

**DOI:** 10.3389/fped.2023.1268971

**Published:** 2023-11-09

**Authors:** Heather L. Young, Clare C. Brown, Brendan Crawford, Richard T. Blaszak, Parthak Prodhan

**Affiliations:** ^1^ Division of Pediatric Infectious Diseases, Department of Pediatrics, University of Arkansas for Medical Sciences, Little Rock, AR, United States; ^2^ Health Policy and Management Department, Fay W. Boozman College of Public Health, University of Arkansas for Medical Sciences, Little Rock, AR, United States; ^3^ Division of Pediatric Nephrology, Department of Pediatrics, University of Arkansas for Medical Sciences, Little Rock, AR, United States; ^4^ Division of Cardiology/Pediatric Critical Care, Department of Pediatrics, University of Arkansas for Medical Sciences, Little Rock, AR, United States

**Keywords:** *Streptococcus pneumoniae*, HUS, hemolytic uremic syndrome, pneumococcus, adverse outcomes

## Abstract

**Introduction:**

Previous small-scale, single-center investigations of *Streptococcus pneumoniae* associated hemolytic uremic syndrome (SpHUS) have shown increased disease severity among SpHUS relative to non-SpHUS patients. Our study compares the impact of *S. pneumoniae* on patient outcomes between SpHUS cases and non-SpHUS controls using the national, multicenter retrospective Pediatric Health Information Systems (PHIS) Database.

**Methods:**

Children <18 years of age with a diagnosis of HUS were included. Univariate analyses and multivariable linear and logistic regressions were utilized to assess the impact of *S. pneumoniae* on mortality, length of stay (LOS), intensive care unit admission (ICU), and mechanical ventilation use. Models were adjusted for demographic and clinical characteristics, including cardiac, neurologic, pulmonary, gastrointestinal, immunologic and renal clinical complications.

**Results:**

Of 3,952 index HUS hospitalizations, 231 (5.8%) were due to SpHUS. SpHUS patients had worse outcomes, including longer hospital stays, increased rate of ICU admission, and increased use of mechanical ventilation (*p* < 0.001 for all). There was a strong positive relationship between clinical complications and adverse outcomes. After adjusting for covariates, SpHUS was associated with an increase in hospital LOS by 3.47 days (*p* = 0.009) and overall ICU-LOS by 4.21 days (*p* < 0.001). SpHUS was also associated with increased likelihood of mechanical ventilation (OR: 3.08; *p* < 0.001), with no increase in ICU admission (*p* = 0.070) and in-hospital mortality (*p* = 0.3874).

**Discussion:**

Our study highlights that SpHUS patients are at increased risk of multiple adverse outcomes likely due to the summative impact of pneumococcal infection and HUS as well as more frequent clinical complications.

## 1. Introduction

Hemolytic uremic syndrome (HUS) consists of a triad of microangiopathic hemolytic anemia, thrombocytopenia, and acute kidney injury occurring after an inciting event causes uncontrolled complement activation. The inciting event can range from medications, genetic disorders, pregnancy, malignancy to more commonly infectious agents: Shiga toxin–producing Escherichia coli (STEC), *Streptococcus (S.) pneumoniae* and human immunodeficiency virus (HIV). Among pediatric cases, Shiga toxin producing E. coli (STEC) is responsible for 90% of all HUS cases, and *S. pneumoniae* infection accounts for almost the entirety of the remainder (1).

While only accounting for a small but sizeable proportion of HUS cases, *S. pneumoniae* associated HUS (SpHUS) is associated with a more severe hospital course and increased morbidity and mortality relative to STEC HUS cases, and more often affect children under the age of 2 years, due to a higher case burden of invasive pneumococcal disease in younger children (2). Despite reductions in pneumococcal infection rates in many countries after conjugated pneumococcal vaccines became part of the primary immunization series, SpHUS rates have continued to increase (3, 4). Children with SpHUS most often have an underlying pneumonia (approximately 65%–92% of cases), with complications including empyema and pleural effusions. In the remainder of cases, meningitis is the presenting focal infection, often with concomitant pneumonia (4–6).

Previous evaluations of SpHUS include small registries or single center retrospective reviews. Our study adds to this important literature by leveraging a multicenter pediatric hospital database spanning 14-years of pediatric hospitalizations. Given that SpHUS is a relatively rare condition, such a large-scale evaluation allows for a more detailed assessment of disease severity and outcomes. Thus, the objective of this study was to characterize the clinical differences between SpHUS and non-SpHUS, and identify risk factors for severe clinical outcomes among patients with SpHUS.

## Materials and methods

This study was deemed non-human subject research by the University of Arkansas for Medical Sciences Institutional Board Review.

### Data

Data for this study came from the Pediatric Health Information System (PHIS) database, years 2004–2018. The PHIS is an administrative hospital discharge database that has discharge data for patients from over 50 not-for-profit pediatric hospitals in the United States. These hospitals, which are associated with the Child Health Corporation, account for approximately 20% of all tertiary care children's hospitals in the United States. The PHIS data includes diagnoses and procedural information using the International Classification of Diseases, Ninth Revision (ICD-9) or Tenth Revision (ICD-10), as well as procedural information using Current Procedural Terminology (CPT) codes.

### Study population

The study sample included index HUS hospitalizations among pediatric patients aged 18 or younger who were discharged from a PHIS-participating hospital with a diagnosis of HUS between January 1, 2004 and December 31, 2018. HUS diagnosis was identified using ICD-9 code 283.11 and ICD-10 code D59.3. Given the varied causes of HUS, we sought to exclude individuals with predispositions for developing HUS (e.g., malignancy, rheumatologic disorders, and pregnancy) as well as individuals with preceding kidney disease. Thus, among the 4,584 index HUS hospitalizations, exclusion of individuals with an ICD-9 code for HUS as well as any concomitant codes for history of chronic kidney disease including nephritic syndrome (*n* = 127), prior plasmapheresis or dialysis (*n* = 74), malignancy (*n* = 90), solid organ transplant (*n* = 63), congenital heart disease (*n* = 93), rheumatologic disease (*n* = 4), pregnancy (*n* = 2), and prior thrombotic microangiopathy or paroxysmal nocturnal hemoglobinuria (*n* = 179) resulted in a final sample of 3,952 patients with index HUS hospitalization.

### Variable definitions

*S. pneumoniae* was defined based on a diagnosis of any pneumococcal condition, including sepsis, meningitis, pneumonia, or infection. Other covariates included demographic variables (age, sex, race) as well as clinical complications and procedures commonly associated with HUS or pneumococcal disease that were identified by their diagnosis codes. We additionally adjusted for an annual linear trend. Clinical complications included the cardiac, neurologic, pulmonary, gastrointestinal, and immunologic complications that are listed in Table 1, as well as sepsis and shock. Primary outcomes for this study were hospital and intensive care unit (ICU) length of stay (LOS) days, and three binary outcomes: in-hospital mortality, utilization of mechanical ventilation, and ICU stay (yes/ no).

**Table 1:** Demographic and clinical characteristics, by SpHUS status.

	Non-SPHUS % (*N*) (*n* = 3,721)	SpHUS % (*N*) (*n* = 231)	*p*-value
Age category			
<1 year	7.9 (293)	12.1 (28)	<0.001
1–5 years	51.3 (1,908)	79.2 (183)	
>5 years	40.8 (1,520)	8.7 (20)	
Sex			
Female	53.1 (1,974)	51.5 (119)	0.65
Male	46.9 (1,747)	48.5 (112)	
Race			
White	77.4 (2,880)	48.5 (112)	<0.001
Black	4.9 (184)	20.8 (48)	
Asian	2.0 (73)	10.4 (24)	
Other	15.7 (584)	20.4 (47)	
Cardiac			
Hypertension	33.7 (1,255)	44.6 (103)	0.001
Pericardial effusion	2.2 (80)	4.8 (11)	0.01
Arrhythmia	0.8 (31)	1.3 (3)	0.457
Congestive heart failure	1.8 (66)	2.2 (5)	0.664
Cardiac arrest	1.2 (43)	4.3 (10)	<0.001
Neurologic			
Seizure	2.8 (105)	4.8 (11)	0.09
Intracranial hemorrhage	0.6 (23)	1.3 (3)	0.214
Brain infarction	2.0 (74)	7.4 (17)	<0.001
Encephalopathy	5.8 (216)	9.1 (21)	0.041
Anoxic brain injury	0.8 (28)	2.2 (5)	0.022
Cerebral edema	0.8 (31)	3.0 (7)	0.001
Meningitis	0.9 (35)	10.0 (23)	<0.001
Pulmonary			
Pneumothorax	0.8 (29)	15.6 (36)	<0.001
Empyema	0.5 (19)	47.2 (109)	<0.001
Pleural effusion	11.2 (416)	65.4 (151)	<0.001
Lung abscess	0.1 (5)	17.7 (41)	<0.001
Gastrointestinal			
Liver disease	3.5 (130)	10.8 (25)	<0.001
Gallbladder disease	1.4 (52)	6.5 (15)	<0.001
Pancreatic disease	14.1 (526)	13.4 (31)	0.762
Peritoneal disease	2.9 (108)	4.3 (10)	0.216
Paralytic ileus	2.7 (101)	5.2 (12)	0.028
Immunodeficiency-Dialysis			
Immunodeficiency	0.7 (26)	2.2 (5)	0.014
Sepsis	8.0 (296)	61.5 (142)	<0.001
Shock	1.9 (72)	4.3 (10)	0.013
Plasmapheresis	4.2 (157)	10.0 (23)	<0.001
Dialysis	38.2 (1,421)	64.9 (150)	<0.001

### Statistical analysis

Descriptive statistics were used to test for differences between demographic and clinical characteristics between patients with and without *S. pneumoniae* using chi-square tests. We compared unadjusted outcomes between patients with and without *S. pneumoniae* using chi square tests and t-tests. To assess the impact of *S. pneumoniae* on patient outcomes, adjusted analyses included linear regressions for LOS variables and logistic regressions for mortality, mechanical ventilation utilization, and any ICU stay. Specifically, we conducted three separate models. First, we adjusted for age, gender, race, and admission year. Second, we additionally adjusted for demographic characteristics, admission year, and complications aggregated into organ systems (i.e., cardiac, neurologic, pulmonary, gastrointestinal, immunologic) in addition to sepsis and shock. Finally, we adjusted for demographic characteristics, admission year, and individual clinical conditions, in addition to the utilization of plasmapheresis or dialysis. In the third model adjusting for individual clinical conditions, we utilized backward stepwise regression with a threshold of *p* < 0.20 and with demographic characteristics and admission year being forced in the analysis. Models included standard errors clustered at the hospital level to account for correlations between outcomes for patients within the same hospital.

This study was deemed non-human subjects research by the University of Arkansas for Medical Sciences Institutional Review Board.

## Results

This study identified 231 SpHUS cases (5.8%) among 3,952 index hospitalizations for HUS. Table 1 depicts the univariate comparison between SpHUS and non-SpHUS groups for demographic and clinical characteristics. Approximately 59% of patients in the non-SpHUS group were less than age 5 years, compared to 91.3% among the SpHUS group (*p* < 0.001). A larger percentage of patients with SpHUS were Black (20.8%), Asian (10.4%), or other race (20.3%) compared to the patients with non-SpHUS (4.9%, 2.0%, and 15.7%, respectively; *p* < 0.001) (Table 1).

SpHUS patients compared to non-SpHUS patients had significantly higher rates of sepsis (61.5% vs. 8.0%, *p* < 0.001); utilization of dialysis (64.9% vs. 38.2%, *p* < 0.001) or plasmapheresis (10.0% vs. 4.2%, *p* < 0.001), as well as neurological, cardiac, and pulmonary complications (Table 1).

In unadjusted analyses, individuals with strep had longer LOS (31.59 days (Standard error [SE]: 1.88_ vs. 15.24 (SE: 0.32 days; *p* < 0.001), longer ICU LOS (14.02 days (SE: 0.98) vs. 3.39 (SE: 0.14) days; *p* < 0.001), higher rates of ICU admission (83.55% vs. 42.38%; *p* < 0.001), higher rates of mortality (6.06% vs. 2.61%; *p* = 0.002), and higher rates of mechanical ventilation use (70.56% vs. 15.42%; *p* > 0.001).

Table 2 provides the association of spHUS with patient outcomes adjusted for patient demographics and admission year. Please see Supplementary Appendix Table S1 for all 95% CIs. spHUS was associated with increased risk of all adverse outcomes, including hospital length of stay (13.68 days; 95: CI: 8.94, 18.42; *p* < 0.001), ICU length of stay (9.75 days; 95: CI: 7.36, 12.14; *p* < 0.001), having ICU admission (aOR: 5.89; 95: CI: 3.28, 10.58; *p* < 0.001), inpatient mortality (aOR: 1.91; 95: CI: 1.05, 3.48; *p* = 0.035), mechanical ventilation (aOR: 12.28; 95: CI: 8.73, 17.27; *p* < 0.001).

**Table 2:** Multivariable regressions adjusted for demographic characteristics and year.

	Hospital LOS (days)		ICU LOS (days)		ICU Admission (Yes/No)		Mortality (Yes/No)		Mechanical Ventilation (Yes/No)	
	Coef (95% CI)	*p*	Coef (95% CI)	*p*	OR (95% CI)	*p*	OR (95% CI)	*p*	OR (95% CI)	*p*
*S. pneumoniae*	13.68	<0.001	9.75	<0.001	5.89	<0.001	1.91	0.035	12.28	<0.001
Admit year	−0.10	0.230	0.01	0.766	1.01	0.351	1.02	0.467	1.05	<0.001
Age										
<1 year	21.41	<0.001	2.71	0.003	0.74	0.091	6.66	<0.001	5.80	<0.001
1–5 years	ref	ref	ref	ref	ref	ref	ref	ref	ref	ref
>5 years	−1.00	0.009	−1.18	<0.001	0.62	<0.001	0.84	0.464	0.64	<0.001
Sex										
Female	ref	ref	ref	ref	ref	ref	ref	ref	ref	ref
Male	0.05	0.917	−0.55	0.049	0.91	0.127	0.99	0.940	0.86	0.099
Race										
White	ref	ref	ref	ref	ref	ref	ref	ref	ref	ref
Black	5.80	0.002	2.27	0.094	1.44	0.059	2.23	0.007	2.24	<0.001
Asian	3.81	0.104	0.18	0.846	0.90	0.638	0.34	0.258	1.31	0.301
Other	1.83	0.046	0.63	0.239	1.21	0.110	1.25	0.324	1.20	0.214

Table 3 provides the association of spHUS adjusted for patient demographic in addition to clinical conditions aggregated by organ systems. SpHUS remained statistically associated with increased risk of ICU admission (aOR: 1.75; 95: CI: 1.01, 3.00; *p* = 0.044) and mechanical ventilation (aOR: 3.50; 95: CI: 2.15, 5.69; *p* < 0.001). Please see Supplementary Appendix Table S2 for all 95% CIs. When adjusting for individual outcomes (Table 4), SpHUS remained associated with increased risk of mechanical ventilation (aOR: 3.21; 95: CI: 1.81, 5.69; *p* < 0.001) (Supplementary Appendix Table S3).

**Table 3:** Multivariable regressions adjusted for demographic characteristics, organ systems, and year.

	Hospital LOS (days)		ICU LOS (days)		ICU Admission (Yes/No)		Mortality (Yes/No)		Mechanical Ventilation (Yes/No)	
	Coef (95% CI)	*p*	Coef (95% CI)	*p*	OR (95% CI)	*p*	OR (95% CI)	*p*	OR (95% CI)	*p*
*S. pneumoniae*	0.76	0.714	2.44	0.081	1.75	0.044	0.63	0.192	3.50	<0.001
Admit year	−0.13	0.131	−0.02	0.687	1.00	0.89	1.00	0.911	1.05	0.003
Age										
<1 year	20.32	<0.001	2.09	0.013	0.62	0.013	5.36	<0.001	8.13	<0.001
1–5 years	ref	Ref	ref	ref	ref	ref	ref	ref	ref	ref
>5 years	−1.41	<0.001	−1.25	<0.001	0.57	<0.001	0.78	0.342	0.49	<0.001
Sex										
Female	ref	ref	ref	ref	ref	ref	ref	ref	ref	ref
Male	−0.09	0.817	−0.68	0.003	0.88	0.055	0.90	0.548	0.79	0.025
Race										
White	ref	ref	ref	ref	ref	ref	ref	ref	ref	ref
Black	3.98	0.053	1.40	0.300	1.34	0.22	1.92	0.033	2.10	0.003
Asian	1.68	0.383	−0.92	0.257	0.74	0.233	0.14	0.045	0.98	0.956
Other	1.40	0.071	0.44	0.364	1.24	0.089	1.19	0.483	1.08	0.659
Organ system complications										
Cardiac	2.62	<0.001	2.23	<0.001	2.82	<0.001	0.89	0.630	1.88	<0.001
Neurologic	7.74	<0.001	4.23	<0.001	3.59	<0.001	4.62	<0.001	8.49	<0.001
Pulmonary	7.16	<0.001	4.63	<0.001	2.89	<0.001	1.89	0.037	3.70	<0.001
Gastrointestinal	10.56	<0.001	3.49	<0.001	2.19	<0.001	0.77	0.316	2.21	<0.001
Immunodeficiency	11.44	0.194	6.36	0.011	3.37	0.068	2.42	0.232	0.98	0.970
Sepsis	11.26	<0.001	5.83	<0.001	2.01	0.001	3.22	<0.001	3.14	<0.001
Shock	4.65	0.187	3.76	0.055	5.25	<0.001	5.84	<0.001	4.71	<0.001

**Table 4:** Multivariable regressions adjusted for demographic characteristics, individual conditions, and year.

	Hospital LOS (days)		ICU LOS (days)		ICU Admission (Yes/No)		Mortality (Yes/No)		Mechanical Ventilation (Yes/No)	
	Coef (95% CI)	*p*	Coef (95% CI)	*p*	OR (95% CI)	*p*	OR (95% CI)	*p*	OR (95% CI)	*p*
*S. pneumoniae* *	−0.78	0.735	1.71	0.192	1.59	0.158	0.57	0.140	3.21	<0.001
Admit year*	−0.01	0.859	0.03	0.349	1.03	0.062	1.00	0.918	1.07	<0.001
Age*										
< 1 year	21.21	<0.001	2.27	0.002	0.77	0.188	6.69	<0.001	9.01	<0.001
1–5 years	ref	ref	ref	ref	ref	ref	ref	ref	ref	ref
> 5 years	−0.85	0.009	−0.98	<0.001	0.58	<0.001	0.82	0.508	0.53	<0.001
Sex*										
Female	ref	ref	ref	ref	ref	ref	ref	ref	ref	ref
Male	0.07	0.855	−0.69	0.001	0.92	0.277	0.85	0.385	0.81	0.052
Race*										
White	ref	ref	ref	ref	ref	ref	ref	ref	ref	ref
Black	4.77	0.026	1.58	0.217	1.70	0.032	2.19	0.013	2.30	<0.001
Asian	1.61	0.356	−0.62	0.423	0.82	0.441	0.25	0.157	0.98	0.951
Other	1.36	0.092	0.49	0.286	1.29	0.066	1.38	0.271	1.08	0.663
Complications										
Hypertension	1.34	0.001	1.22	<0.001	2.21	<0.001	0.52	0.029	1.24	0.057
Pericardial effusion	3.63	0.157	2.34	0.043	2.00	0.048			1.87	0.057
Arrhythmia			3.05	0.197					1.99	0.139
Congestive heart failure	4.70	0.107	5.52	0.004	2.56	0.026			2.44	0.017
Cardiac arrest			7.74	0.125	3.63	0.005	28.87	<0.001	6.84	0.001
Seizure			1.66	0.105	2.40	0.002			3.38	<0.001
Intracranial hemorrhage			8.75	0.047	3.19	0.024	3.94	0.005	10.53	0.001
Brain infarction	4.75	0.081	4.09	0.032	3.59	0.001	6.02	0.001	1.93	0.183
Encephalopathy	8.03	<0.001	3.70	<0.001	3.27	<0.001	0.41	0.084	5.25	<0.001
Anoxic brain injury	19.70	0.043			0.55	0.197	2.96	0.026		
Cerebral edema			−3.04	0.165	3.16	0.042	10.97	<0.001	3.34	0.025
Meningitis									6.20	0.019
Pneumothorax	10.73	0.047	10.95	0.028					1.70	0.179
Empyema					1.48	0.191			1.66	0.12
Pleural effusion	3.52	<0.001	2.56	<0.001	2.38	<0.001	1.64	0.142	2.82	<0.001
Lung abscess	12.80	0.008	4.22	0.164						
Liver disease	7.45	0.012	4.10	0.055	1.45	0.187			1.45	0.096
Gallbladder disease	8.21	0.02							1.65	0.112
Pancreatic disease	5.62	<0.001	1.10	0.036	1.53	0.008				
Peritoneal disease	14.31	0.001	5.12	<0.001	1.74	0.155			2.47	0.001
Paralytic ileus	7.77	0.006	4.46	0.004	2.07	0.008			2.98	0.001
Immunodeficiency			4.76	0.016	3.50	0.09	3.84	0.124		
Sepsis	10.79	<0.001	4.76	<0.001	1.78	0.005	3.26	0.001	2.71	<0.001
Shock					5.28	0.001	3.11	0.021	3.63	0.004
Plasmapheresis			1.81	0.128	1.69	0.096			1.97	0.039
Dialysis	4.06	<0.001	1.99	<0.001	3.20	<0.001	0.66	0.174	2.18	<0.001

* Forced to be included in model regardless of *p*-value.

## Discussion

This investigation, which leveraged a large multicenter hospital database, is the largest systematic assessment of SpHUS and confirms that patients with SpHUS have a much more severe clinical course than non-SpHUS cases, indicated by the increased presence of clinical complications among SpHUS patients compared to non-SpHUS patients. These complications in turn are associated with worse outcomes and may be indicative of disease severity. One study that examined data from 1997 to 2009 showed that patients with SpHUS compared to non-SpHUS patients had increased rates of dialysis, increased lengths of stay and total hospital charges but did not include other measures of severity (2).

*S. pneumoniae* can cause significant invasive disease in children, with pneumonia being the most common manifestation. Pneumonia can be complicated by pleural effusions, empyema, sepsis, and can require significant supportive care. After pneumonia, meningitis is the second most common form of *S. pneumoniae* presenting with HUS (6). While any bacterial meningitis can be profoundly devastating, *S. pneumoniae* meningitis is associated with more severe disease and mortality than other causes of bacterial meningitis, and is often complicated by stroke and subdural empyema (7, 8). Thus it is not surprising that in our study, SpHUS cases were associated with having multiple complications, especially those related to sepsis, respiratory complications, and neurologic complications. The worse outcomes in SpHUS patients may be indicative of a summative impact of invasive pneumococcal disease and renal involvement from HUS.

Dialysis was utilized in 65% of SpHUS patients compared to 38% in non-SpHUS patients. The higher rate of dialysis use among SpHUS patients is similar to those reported previously in small case series whereby over 70% of SpHUS patients required dialysis even in the era of universal 13-valent pneumococcal vaccination (5, 7, 9–12). A study of 12 patients with SpHUS compared to 17 non-SpHUS patients similarly found a dialysis rate of 75% in SpHUS patients. (9) Overall, our results, are novel in terms of sample size and re-affirm findings of previous small case series that have indicated that SpHUS patients tend to have more need for dialysis compared to non-SpHUS patients. Our study shows increased use of plasmapheresis in the SpHUS population compared to non-SpHUS. The proposed pathophysiologic mechanism involving neuraminidase release and subsequent activation of the Thomsen- Friedenreich antigen has led some to advocate for plasmapheresis in management. (13) Several case reports have highlighted potential benefit in severe SpHUS disease (14–16). Currently, plasmapheresis is not universally recommended as a standard treatment (17). The current study highlights the utilization of plasmapheresis but fully measuring the efficacy of plasmapheresis as a treatment modality was out of scope of this study.

There are several limitations to our study. This study used an administrative dataset that was not collected specifically for research purposes. The use of the PHIS administrative database offers the advantage of a large samples size, but it is limited by the fact that specific data points are not available, such as laboratory values or mortality outside of the acute inpatient setting**.** Follow-up after the primary hospitalization for chronic sequelae was not performed in our study cohort which excluded analysis for long-term morbidities. Relatedly, we were also unable to quantify the severity of lung disease from the variables available to us. We had no access to vaccination status or *S. pneumoniae* serotype data to evaluate the role that vaccination has played or the emergence of various serotypes that may be more prone to induce HUS. Furthermore, as the pathogenic mechanisms behind all forms of HUS are evolving, so is the categorization of HUS. We were able to identify individuals with a diagnosis code for HUS and then excluded individuals with pre-existing conditions that would make them more prone to develop a secondary HUS as well as have other confounding factors that would make them more at risk of complications. However, our efforts may not have removed all causes of secondary HUS and thus we represented our data as SpHUS and nonSpHUS to distinguish these caveats. Furthermore, we only identified the selected subset of pneumococcal virus with the defined ICD codes. Despite these limitations, this study provides the distinctive advantage of evaluating a large-scale sample of pediatric data from many United States metropolitan areas and can serve as a foundation for clinical implications as well as for further investigation through a prospective study design. Future studies should additionally consider outcomes after the index hospitalization, such as readmission to the hospital.

Our study confirms that SpHUS is more severe than non-SpHUS. The worse outcomes in SpHUS patients may be indicative of a summative impact of multiple co-morbidities from invasive pneumococcal disease and renal involvement from HUS. These complications may have long term implications, but it is beyond the scope of this study to investigate these. The role of vaccination and *S. pneumoniae* serotypes would also benefit from further study.

## Data Availability

The data analyzed in this study is subject to the following licenses/restrictions: This is the Pediatric Health Information System® Database that is only available to Children's Hospital Association partners and is not publicly available. Requests to access these datasets should be directed to analytics@childrenshospitals.org.

## References

[1] Aigner C Schmidt A Gaggl M Sunder-Plassmann G. An updated classification of thrombotic microangiopathies and treatment of complement gene variant-mediated thrombotic microangiopathy. Clin Kidney J. (2019) 12(3):333–7. 10.1093/ckj/sfz040 31198225 PMC6543965

[2] Veesenmeyer AF Edmonson MB. Trends in US hospital stays for Streptococcus pneumoniae-associated hemolytic uremic syndrome. Pediatr Infect Dis J. (2013) 32(7):731–5. 10.1097/INF.0b013e31828b31c8 23838775

[3] Matanock A Lee G Gierke R Kobayashi M Leidner A Pilishvili T. Use of 13-valent pneumococcal conjugate vaccine and 23-valent pneumococcal polysaccharide vaccine among adults aged ≥65 years: updated recommendations of the advisory committee on immunization practices. MMWR Morb Mortal Wkly Rep. (2019) 68(46):1069–75. 10.15585/mmwr.mm6846a5 31751323 PMC6871896

[4] Bender JM Ampofo K Byington CL Grinsell M Korgenski K Daly JA Epidemiology of Streptococcus pneumoniae-induced hemolytic uremic syndrome in Utah children. Pediatr Infect Dis J. (2010) 29(8):712–6. 10.1097/INF.0b013e3181db03a7 20661100

[5] Banerjee R Hersh AL Newland J Beekmann SE Polgreen PM Bender J Streptococcus pneumoniae-associated hemolytic uremic syndrome among children in North America. Pediatr Infect Dis J. (2011) 30(9):736–9. 10.1097/INF.0b013e3182191c58 21772230

[6] Makwana A Sheppard C Fry NK Ladhani SN. Pneumococcal-related hemolytic uremic syndrome in the United Kingdom: national surveillance, 2006–2016. Pediatr Infect Dis J. (2019) 38(10):e254–9. 10.1097/INF.0000000000002368 31232894

[7] Nathanson S Deschênes G. Prognosis of Streptococcus pneumoniae-induced hemolytic uremic syndrome. Pediatr Nephrol. (2001) 16(4):362–5. 10.1007/s004670100564 11354782

[8] Baraff LJ Lee SI Schriger DL. Outcomes of bacterial meningitis in children: a meta-analysis. Pediatr Infect Dis J. (1993) 12(5):389–94. 10.1097/00006454-199305000-00008 8327300

[9] Brandt J Wong C Mihm S Roberts J Smith J Brewer E Invasive pneumococcal disease and hemolytic uremic syndrome. Pediatrics. (2002) 110(2 Pt 1):371–6. 10.1542/peds.110.2.371 12165593

[10] Cabrera GR Fortenberry JD Warshaw BL Chambliss CR Butler JC Cooperstone BG. Hemolytic uremic syndrome associated with invasive Streptococcus pneumoniae infection. Pediatrics. (1998) 101(4 Pt 1):699–703. 10.1542/peds.101.4.699 9521959

[11] Waters AM Kerecuk L Luk D Haq MR Fitzpatrick MM Gilbert RD Hemolytic uremic syndrome associated with invasive pneumococcal disease: the United Kingdom experience. J Pediatr. (2007) 151(2):140–4. 10.1016/j.jpeds.2007.03.055 17643764

[12] Lawrence J Gwee A Quinlan C. Pneumococcal haemolytic uraemic syndrome in the postvaccine era. Arch Dis Child. (2018) 103(10):957–61. 10.1136/archdischild-2017-313923 29674516

[13] Cochran JB Panzarino VM Maes LY Tecklenburg FW. Pneumococcus-induced T-antigen activation in hemolytic uremic syndrome and anemia. Pediatr Nephrol. (2004) 19(3):317–21. 10.1007/s00467-003-1382-z 14714171

[14] Petras ML Dunbar NM Filiano JJ Braga MS Chobanian MC Szczepiorkowski ZM. Therapeutic plasma exchange in Streptococcus pneumoniae-associated hemolytic uremic syndrome: a case report. J Clin Apher. (2012) 27(4):212–4. 10.1002/jca.21208 22307916

[15] Weintraub L Ahluwalia M Dogra S Uehlinger J Skversky A Vasovic L. Management of streptococcal pneumoniae-induced hemolytic uremic syndrome: a case report. Clin Nephrol Case Stud. (2014) 2:9–17. 10.5414/CNCS107887 29043123 PMC5437990

[16] Hopkins CK Yuan S Lu Q Ziman A Goldfinger D. A severe case of atypical hemolytic uremic syndrome associated with pneumococcal infection and T activation treated successfully with plasma exchange. Transfusion. (2008) 48(11):2448–52. 10.1111/j.1537-2995.2008.01871.x 18673349

[17] Spinale JM Ruebner RL Kaplan BS Copelovitch L. Update on Streptococcus pneumoniae associated hemolytic uremic syndrome. Curr Opin Pediatr. (2013) 25(2):203–8. 10.1097/MOP.0b013e32835d7f2c 23481474

