# Supplementary material for: Streptococcus pneumoniae associated hemolytic uremic syndrome in children
Source: Front Pediatr. 2023 Nov 9;11:1268971. doi: 10.3389/fped.2023.1268971 (PMC10665843; doi:10.3389/fped.2023.1268971)
Supplement: Supplementary file 1 [file Datasheet1.pdf]

Appendix Table 1 Multivariable regressions adjusted for demographic characteristics and year

	Hospital LOS (days)		ICU LOS (days)		ICU Admission (Yes/No)		Mortality (Yes/No)		Mechanical Ventilation (Yes/No)	
	Coef (95% CI)	p	Coef (95% CI)	p	OR (95% CI)	p	OR (95% CI)	p	OR (95% CI)	p
S. pneumoniae	13.68 (8.94, 18.42)	<0.001	9.75 (7.36, 12.14)	<0.001	5.89 (3.28, 10.58)	<0.001	1.91 (1.05, 3.48)	<0.001	12.28 (8.73, 17.27)	<0.001
Age										
< 1 year	21.41 (14.71, 28.11)	<0.001	2.71 (0.99, 4.43)	<0.001	0.74 (0.52, 1.05)	<0.001	6.66 (3.72, 11.90)	<0.001	5.80 (4.42, 7.62)	<0.001
1-5 years	ref	ref	ref	ref	ref	ref	ref	ref	ref	ref
> 5 years	-1.00 (-1.74, -0.26)	0.009	-1.18 (-1.72, -0.64)	0.009	0.62 (0.54, 0.71)	0.009	0.84 (0.53, 1.34)	0.009	0.64 (0.50, 0.81)	0.009
Sex										
Female	ref	ref	ref	ref	ref	ref	ref	ref	ref	ref
Male	0.05 (-0.96, 1.06)	0.917	-0.55 (-1.09, 0.00)	0.917	0.91 (0.81, 1.03)	0.917	0.99 (0.70, 1.40)	0.917	0.86 (0.71, 1.03)	0.917
Race										
White	ref	ref	ref	ref	ref	ref	ref	ref	ref	ref
Black	5.80 (2.29, 9.30)	0.002	2.27 (-0.40, 4.93)	0.002	1.44 (0.99, 2.09)	0.002	2.23 (1.25, 3.99)	0.002	2.24 (1.54, 3.25)	0.002
Asian	3.81 (-0.82, 8.43)	0.104	0.18 (-1.68, 2.04)	0.104	0.90 (0.59, 1.39)	0.104	0.34 (0.05, 2.21)	0.104	1.31 (0.78, 2.20)	0.104
Other	1.83 (0.03, 3.63)	0.046	0.63 (-0.43, 1.69)	0.046	1.21 (0.96, 1.53)	0.046	1.25 (0.80, 1.96)	0.046	1.20 (0.90, 1.60)	0.046
Admit year	-0.10 (-0.27, 0.07)	0.230	0.01 (-0.08, 0.11)	0.23	1.01 (0.99, 1.04)	0.230	1.02 (0.97, 1.07)	0.230	1.05 (1.02, 1.08)	0.230

Appendix Table 2 Multivariable regressions adjusted for demographic characteristics, organ systems, and year

	Hospital LOS (days)		ICU LOS (days)		ICU Admission (Yes/No)		Mortality (Yes/No)		Mechanical Ventilation (Yes/No)	
	Coef (95% CI)	p	Coef (95% CI)	p	OR (95% CI)	p	OR (95% CI)	p	OR (95% CI)	p
S. pneumoniae	0.76 (-3.38, 4.90)	0.714	2.44 (-0.32, 5.20)	0.081	1.75 (1.01, 3.00)	0.044	0.63 (0.31, 1.26)	0.192	3.50 (2.15, 5.69)	<0.001
Age										
< 1 year	20.32 (13.67, 26.97)	<0.001	2.09 (0.46, 3.73)	0.013	0.62 (0.43, 0.90)	0.013	5.36 (2.86, 10.05)	<0.001	8.13 (5.42, 12.20)	<0.001
1-5 years	ref	Ref	ref	ref	ref	ref	ref	ref	ref	Ref
> 5 years	-1.41 (-2.05, -0.76)	<0.001	-1.25 (-1.69, -0.82)	<0.001	0.57 (0.48, 0.66)	<0.001	0.78 (0.47, 1.30)	0.342	0.49 (0.38, 0.63)	<0.001
Sex										
Female	ref	ref	ref	ref	ref	ref	ref	ref	ref	ref
Male	-0.09 (-0.84, 0.67)	0.817	-0.68 (-1.12, -0.24)	0.003	0.88 (0.77, 1.00)	0.055	0.90 (0.63, 1.28)	0.548	0.79 (0.64, 0.97)	0.025
Race										
White	ref	ref	ref	ref	ref	ref	ref	ref	ref	ref
Black	3.98 (-0.05, 8.01)	0.053	1.40 (-1.28, 4.07)	0.3	1.34 (0.84, 2.15)	0.22	1.92 (1.05, 3.51)	0.033	2.10 (1.28, 3.44)	0.003
Asian	1.68 (-2.15, 5.50)	0.383	-0.92 (-2.52, 0.69)	0.257	0.74 (0.45, 1.21)	0.233	0.14 (0.02, 0.96)	0.045	0.98 (0.48, 2.02)	0.956
Other	1.40 (-0.12, 2.93)	0.071	0.44 (-0.52, 1.40)	0.364	1.24 (0.97, 1.60)	0.089	1.19 (0.73, 1.96)	0.483	1.08 (0.76, 1.55)	0.659
Cardiac	2.62 (1.54, 3.70)	<0.001	2.23 (1.66, 2.79)	<0.001	2.82 (2.38, 3.34)	<0.001	0.89 (0.56, 1.41)	0.63	1.88 (1.53, 2.30)	<0.001
Neurologic	7.74 (4.94, 10.54)	<0.001	4.23 (2.94, 5.52)	<0.001	3.59 (2.47, 5.23)	<0.001	4.62 (2.60, 8.21)	<0.001	8.49 (5.63, 12.82)	<0.001
Pulmonary	7.16 (5.39, 8.93)	<0.001	4.63 (3.27, 5.99)	<0.001	2.89 (2.09, 3.99)	<0.001	1.89 (1.04, 3.43)	0.037	3.70 (2.80, 4.88)	<0.001

Gastrointestinal	10.56 (8.76, 12.36)	<0.001	3.49 (2.40, 4.58)	<0.001	2.19 (1.74, 2.76)	<0.001	0.77 (0.46, 1.28)	0.316	2.21 (1.63, 3.00)	<0.001
Immunodeficiency	11.44 (-6.01, 28.89)	0.194	6.36 (1.50, 11.23)	0.011	3.37 (0.91, 12.43)	0.068	2.42 (0.57, 10.30)	0.232	0.98 (0.37, 2.63)	0.97
Sepsis	11.26 (8.49, 14.03)	<0.001	5.83 (3.92, 7.75)	<0.001	2.01 (1.34, 3.00)	0.001	3.22 (1.75, 5.91)	<0.001	3.14 (2.21, 4.45)	<0.001
Shock	4.65 (-2.33, 11.62)	0.187	3.76 (-0.08, 7.59)	0.055	5.25 (2.22, 12.40)	<0.001	5.84 (2.86, 11.94)	<0.001	4.71 (2.05, 10.84)	<0.001
Admit year	-0.13 (-0.29, 0.04)	0.131	-0.02 (-0.09, 0.06)	0.687	1.00 (0.98, 1.03)	0.89	1.00 (0.94, 1.06)	0.911	1.05 (1.02, 1.09)	0.003

Appendix Table 3 Multivariable regressions adjusted for demographic characteristics, individual conditions, and year

	Hospital LOS (days)		ICU LOS (days)		ICU Admission (Yes/No)		Mortality (Yes/No)		Mechanical Ventilation (Yes/No)	
	Coef (95% CI)	p	Coef (95% CI)	p	OR (95% CI)	p	OR (95% CI)	p	OR (95% CI)	p
S. pneumoniae[a]	-0.78 (-5.41, 3.84)	0.735	1.71 (-0.89, 4.32)	0.192	1.59 (0.84, 3.02)	0.158	0.57 (0.27, 1.20)	0.14	3.21 (1.81, 5.69)	<0.001
Age[a]										
< 1 year	21.21 (14.66, 27.77)	<0.001	2.27 (0.85, 3.70)	0.002	0.77 (0.53, 1.13)	0.188	6.69 (3.25, 13.77)	<0.001	9.01 (6.04, 13.44)	<0.001
1-5 years	ref	ref	ref	ref	ref	ref	ref	ref	ref	ref
> 5 years	-0.85 (-1.47, -0.22)	0.009	-0.98 (-1.40, -0.55)	<0.001	0.58 (0.50, 0.69)	<0.001	0.82 (0.46, 1.47)	0.508	0.53 (0.40, 0.70)	<0.001
Sex[a]										
Female	ref	ref	ref	ref	ref	ref	ref	ref	ref	ref
Male	0.07 (-0.66, 0.79)	0.855	-0.69 (-1.09, -0.29)	0.001	0.92 (0.79, 1.07)	0.277	0.85 (0.58, 1.23)	0.385	0.81 (0.66, 1.00)	0.052
Race[a]										
White	ref	ref	ref	ref	ref	ref	ref	ref	ref	ref
Black	4.77 (0.59, 8.95)	0.026	1.58 (-0.96, 4.12)	0.217	1.70 (1.05, 2.75)	0.032	2.19 (1.18, 4.08)	0.013	2.30 (1.44, 3.67)	<0.001
Asian	1.61 (-1.86, 5.08)	0.356	-0.62 (-2.17, 0.92)	0.423	0.82 (0.49, 1.37)	0.441	0.25 (0.04, 1.69)	0.157	0.98 (0.48, 2.00)	0.951
Other	1.36 (-0.23, 2.94)	0.092	0.49 (-0.43, 1.41)	0.286	1.29 (0.98, 1.68)	0.066	1.38 (0.78, 2.45)	0.271	1.08 (0.75, 1.56)	0.663
Admit year[a]	-0.01 (-0.18, 0.15)	0.859	0.03 (-0.04, 0.10)	0.349	1.03 (1.00, 1.06)	0.062	1.00 (0.93, 1.07)	0.918	1.07 (1.03, 1.11)	<0.001
Hypertension	1.34 (0.56, 2.11)	0.001	1.22 (0.65, 1.78)	<0.001	2.21 (1.83, 2.66)	<0.001	0.52 (0.29, 0.94)	0.029	1.24 (0.99, 1.55)	0.057

Pericardial effusion	3.63 (-1.44, 8.70)	0.157	2.34 (0.08, 4.60)	0.043	2.00 (1.00, 3.99)	0.048			1.87 (0.98, 3.57)	0.057
Arrhythmia			3.05 (-1.63, 7.72)	0.197					1.99 (0.80, 4.94)	0.139
Congestive heart failure	4.70 (-1.05, 10.44)	0.107	5.52 (1.83, 9.21)	0.004	2.56 (1.12, 5.86)	0.026			2.44 (1.17, 5.10)	0.017
Cardiac arrest			7.74 (-2.22, 17.71)	0.125	3.63 (1.48, 8.92)	0.005	28.87 (11.42, 72.99)	<0.001	6.84 (2.26, 20.68)	0.001
Seizure			1.66 (-0.36, 3.68)	0.105	2.40 (1.39, 4.14)	0.002			3.38 (1.95, 5.88)	<0.001
Intracranial hemorrhage			8.75 (0.11, 17.40)	0.047	3.19 (1.16, 8.72)	0.024	3.94 (1.51, 10.33)	0.005	10.53 (2.70, 41.05)	0.001
Brain infarction	4.75 (-0.61, 10.12)	0.081	4.09 (0.36, 7.81)	0.032	3.59 (1.64, 7.84)	0.001	6.02 (2.09, 17.29)	0.001	1.93 (0.73, 5.09)	0.183
Encephalopathy	8.03 (4.78, 11.28)	<0.001	3.70 (1.95, 5.45)	<0.001	3.27 (1.87, 5.74)	<0.001	0.41 (0.15, 1.13)	0.084	5.25 (3.05, 9.04)	<0.001
Anoxic brain injury	19.70 (0.66, 38.74)	0.043			0.55 (0.22, 1.37)	0.197	2.96 (1.14, 7.67)	0.026		
Cerebral edema			-3.04 (-7.37, 1.29)	0.165	3.16 (1.04, 9.54)	0.042	10.97 (3.53, 34.14)	<0.001	3.34 (1.17, 9.58)	0.025
Meningitis									6.20 (1.34, 28.56)	0.019
Pneumothorax	10.73 (0.16, 21.31)	0.047	10.95 (1.26, 20.63)	0.028					1.70 (0.78, 3.68)	0.179
Empyema					1.48 (0.82, 2.65)	0.191			1.66 (0.88, 3.16)	0.12

Pleural effusion	3.52 (1.66, 5.38)	<0.001	2.56 (1.23, 3.90)	<0.001	2.38 (1.78, 3.19)	<0.001	1.64 (0.85, 3.19)	0.142	2.82 (2.04, 3.89)	<0.001
Lung abscess	12.80 (3.51, 22.08)	0.008	4.22 (-1.77, 10.21)	0.164						
Liver disease	7.45 (1.72, 13.18)	0.012	4.10 (-0.10, 8.30)	0.055	1.45 (0.84, 2.50)	0.187			1.45 (0.94, 2.24)	0.096
Gallbladder disease	8.21 (1.37, 15.05)	0.02							1.65 (0.89, 3.05)	0.112
Pancreatic disease	5.62 (3.47, 7.77)	<0.001	1.10 (0.07, 2.13)	0.036	1.53 (1.12, 2.09)	0.008				
Peritoneal disease	14.31 (5.78, 22.85)	0.001	5.12 (2.59, 7.66)	<0.001	1.74 (0.81, 3.72)	0.155			2.47 (1.45, 4.21)	0.001
Paralytic ileus	7.77 (2.28, 13.26)	0.006	4.46 (1.52, 7.39)	0.004	2.07 (1.21, 3.54)	0.008			2.98 (1.58, 5.62)	0.001
Immunodeficiency			4.76 (0.91, 8.61)	0.016	3.50 (0.82, 14.87)	0.09	3.84 (0.69, 21.39)	0.124		
Sepsis	10.79 (7.47, 14.12)	<0.001	4.76 (3.19, 6.33)	<0.001	1.78 (1.19, 2.66)	0.005	3.26 (1.60, 6.66)	0.001	2.71 (1.88, 3.90)	<0.001
Shock					5.28 (2.05, 13.62)	0.001	3.11 (1.19, 8.13)	0.021	3.63 (1.51, 8.72)	0.004
Plasmapheresis			1.81 (-0.54, 4.15)	0.128	1.69 (0.91, 3.14)	0.096			1.97 (1.03, 3.77)	0.039
Dialysis	4.06 (2.91, 5.22)	<0.001	1.99 (1.29, 2.68)	<0.001	3.20 (2.47, 4.14)	<0.001	0.66 (0.36, 1.20)	0.174	2.18 (1.69, 2.82)	<0.001

[a] *Forced to be included in model regardless of p-value.*
